# A Review of the Health Implications of Heavy Metals in Food Chain in Nigeria

**DOI:** 10.1155/2020/6594109

**Published:** 2020-04-16

**Authors:** Ugonna C. Nkwunonwo, Precious O. Odika, Nneka I. Onyia

**Affiliations:** ^1^Department of Geoinformatics and Surveying, University of Nigeria, Enugu Campus, Nsukka, Nigeria; ^2^Department of Geological Sciences, Nnamdi Azikiwe University, Awka, Nigeria; ^3^Faculty of Environmental Sciences, Enugu State University of Science and Technology, Enugu, Nigeria; ^4^Women's Union, Diocese of Nike, Church of Nigeria, Anglican Communion, Enugu, Nigeria

## Abstract

Heavy metals such as Zn, Pb, Fe, and Cu are abundant in the environment and contribute largely to the sustainability and equilibrium of ecosystem processes. However, because of their bioaccumulation, nondegradability, and the excessive amounts in which they exist, these metals contaminate the food chain and subsequently become a source of toxicity to human beings and the entire ecological function. This is a major issue of concern within the study of environmental science and geochemistry. Although there is a global significance to the issue, it seems more immediate for the developing countries (DCs) such as Nigeria, where the pressure of the teeming population escalates the exigency for human sustainability, food security, and total eradication of hunger. Within the Nigerian context, many studies have examined this all-important issue, but most of these studies are fragmented and limited within the purview of mostly individual states and localities within the country. Taken on a wider geographical scale, the discussions and perspectives of these studies on heavy metal contamination of the food chain offer insufficient insight and expose merely a snapshot of the actual situation. As a result of this, a country-wide knowledge base of the implications of heavy metals on the food chain is lacking. Thus, the present study synthesises existing literature and their findings to create a knowledge base on the vulnerability of the food chain in Nigeria. Aquatic foods, fruits, vegetables, and major staple food such as tubers are the major host of carcinogenic and mutagenic components of heavy metals in Nigeria. This study motivates the Standard Organisation of Nigeria (SON), along with other food and agricultural agencies, to intensify their efforts in monitoring and analysing food components, and we advise consumers to eat with certain degrees of caveat.

## 1. Introduction

Since his famous publication titled, “*What is a heavy metal?*,” Hawkes [1] has spurred far greater interests for more and more debates within environmental science, geochemistry, and toxicology research. Although Hawkes' study, from the stand point of natural and pure sciences research, primarily constructed a theoretical paradigm that lays the foundation for a critical understanding of heavy metals, it has definitely instigated a rising academic discussion, which now focuses on the abundance and implications of heavy metals within the human-ecological systems. Hawkes' study is also a seminal tool to drive a deeper and more cognate exploration of heavy metals. The context by which he introduced and expounded heavy metals as the block of metals and metalloids found in Groups 3 to 16 and in Periods 4 and greater of the periodic table makes sense to conclude that a metal is heavy not necessarily because of its density, rather its chemistry. This has remained a building block which various studies, in environmental science literature, have used to define heavy metals comprising metals and metalloids which are associated with adverse environmental effects, mostly pollution, toxicity, and contamination. Still, the diversity of definitions accorded to heavy metals in the literature creates an ideological conflict because of the dominance of density, relative atomic mass, and atomic number in the conceptualisation of heavy metals. Recently, Ali and Khan [2] argued on a more comprehensive idea which suggests that heavy metals are naturally occurring metals having an atomic number greater than 20 and an elemental density greater than 5 gcm^−3^. The study provided a list of fifty one elements that it referred to as heavy metals—with the exclusion of tin and arsenic. Obviously, there is an active debate on the conceptualisations of heavy metals which, needless to say, is not the focus of this study. However, many ideas drawn from extant research are logical pointers to what might be the likely implications of heavy metals on the food chain.

Basically, heavy metals are unarguably the transition and posttransition metals, and the examples which are common in various literature are lead (Pb), cadmium (Cd), vanadium (V), cobalt (Co), chromium (Cr), copper (Cu), iron (Fe), arsenic (As), nickel (Ni), manganese (Mn), tin (Sn), zinc (Zn), and mercury (Hg). The availability and accessibility of these metals and metalloids through natural and anthropogenic pathways remain a major global concern in the ecosystem [3–5]. The sources of heavy metals in the surface environment are natural and anthropogenic. Natural sources include parent rocks and metallic minerals. Anthropogenic sources include agriculture (fertilizers, pesticides, etc.), metallurgy (mining, smelting etc.), energy production (power plant, leaded gasoline, etc.), and sewage disposal [6, 7]. A study at Ishiagu in Nigeria showed that while the Pb-Zn deposit is the natural source of heavy metals in the area, anthropogenic sources (from the use of power plants) could also be significant as power plants have been up to the time of the study the only source of electricity in the area [7]. In the North China Plain, the application of farmyard and chemical fertilizer was the pathway through which some heavy metals migrated into the soil [8]. In assessing the sources of heavy metals in the surface environment in a study area, anthropogenic activities of the habitants remain a major factor. In Nigeria, mining of ore deposits, oil and gas exploration and exploitation, and agriculture, besides manufacturing, construction, and production activities are among the several commercial and artisanal extractions of natural resources, capable of influencing the disruption, dispersion, and distribution of naturally placed heavy metals and metalloid to the environment [9]. These metals and metalloid are further influenced by the conditions of the environment of deposition such as acidity, alkalinity, pH, adsorption, speciation, and temperature which inform their solubility, mobility, availability, and accessibility. They also make their way into the different environmental media such as soil, water, rock, and sediment from which we grow most of our food.

The chemistry of heavy metals is a major contribution to their implications within the human-ecological context. In terms of usefulness, the universe had been largely blessed by the abundance of heavy metals and the roles they play both in keeping the equilibrium and sustainability of ecosystem functions [10–12]. For example, Fe which is the most abundant metal on Earth and very electropositive can combine easily with O2 (oxygen gas) and thus forms a rich compound for industrial and biological purposes [13]. In their experimental research, Bae et al. [14] assiduously showed the crucial roles that Zn plays in dermatology. Cu is effectively used in the treatment for fish diseases [15] and to treat radiation sickness [16]. Nagajyoti et al. [17] argued that in trace amounts, most of the heavy metals are of significance to plant metabolism. However, these metals and metalloids adversely affect lives in the ecosystem, including humans, plants, and animals through their bioaccumulation and bioaugmentation in the food chain [18, 19]. Alkorta et al. [20] and Volesky [21] were of the opinion that most heavy metals are toxic even at low concentration of about 0.1 to 0.3 mg/l. However, pH level plays a vital role in heavy metal ion adsorption from an aqueous solution, and speciation and adsorption level influence the mobility of these metals [22]. The soil type can also influence adsorption of heavy metals; for instance, soil clay minerals have a greater influence on adsorption and inactivation compared with soil organic matter [23].

In view of these impacts, Ali et al. [24] observed that some heavy metals possess the ability to disrupt metabolic activities and genetic makeup, while others affect embryonic or foetal development. This often results in cancer, developmental disorders, and neurological and behavioural changes often found in children. So, the health impacts of heavy metals on the food chain, which include the soil, plants, and aquatic life, cannot be more obvious. As a major source of human exposure to heavy metal toxicity, the food chain is a critical component that now draws the attention of major discussions within the academy of nutritional and environmental sciences (see, for example, [25–27]). With the increasing human populations and rising issues of food insecurity which have heightened the demand for genetically engineered food, it is now ever more crucial that current research should examine the typology of heavy metals found in the food chain and the pathway in which these metals contaminate a range of staple foods consumed by many people within the diversity of geographical contexts.

This study focuses on the negative impacts of heavy metals and metalloids in the food chain regarding their total concentration levels in the environment in Nigeria. With the early industrialisation stage in Nigeria and poor implementation of environmental protection policies and regulations, its environmental pollution and contamination have been a great concern. According to Onakpa et al. [28], both natural and anthropogenic sources are pathways by which heavy metals and metalloids contaminate many Nigeria food crops and vegetables. Previous studies on the health implications of heavy metals are fragmented and mostly focused on individual states and localities within Nigeria [29–31]. The findings of these studies are consistent with the general view that heavy metals are nonbiodegradable, and metallic elements with relatively high density are toxic or poisonous even at low concentrations. They accumulate in the environment and over time contaminate the food chain, which is a major source of environmental and human health risk [1, 24, 32, 33]. Despite the obvious significance of these studies, a country-wide knowledge base of the implication of heavy metals on the food chain within different regional and socioeconomic settings of Nigeria is lacking. This is a major gap in science, which this review makes efforts to address. It provides important insight into the toxicity of heavy metals and the particular foods that are increasingly vulnerable to heavy metals and hazardous for consumption with respect to different geographical regions in Nigeria. The authors synthesise the findings of the current studies to create a knowledge base which somewhat simplifies an understanding of the impacts of heavy metals on the food chain within the context of Nigeria. Given the high population of Nigeria, its extreme poverty level, and environmental and public health challenges [34], food availability and consumption are key factors to its continuous existence. Therefore, research must inform decision towards ensuring that health and sustainability are core in the choice of foods that Nigerians consume. This will mitigate several health issues that constrain economic and human capital development within the country.

## 2. Methods and Data

To achieve the objectives of this review, the authors conducted a structured search process to identify the body of the current literature relevant to the study. In collecting relevant literature, the authors ensured that they maintained quality and academic standard, which are often the basis of most literature review studies, for example, Manikas and Hansen [35]; Nkwunonwo et al. [36]; Thonemann and Schumann [37]. Therefore, the present literature search considered mainly research articles published in highly reputed journals (indexed by Scopus and Scimago and ranked in Thompson Reuter). The authors consulted EBSCO's environmental database, which hosts top open access environmental research journals: Greenfile, which archives well-researched debates covering all aspects of human impact on the environment. The study also considered several other libraries including DOAJ (Directory of Open Access Journals) digital Library, Google Scholar, PubMed, E-resources, IEEE Explore, SpringerLink, ScienceDirect, and Thomson Reuters' Web of Science. Key terms such as “heavy metals,” “food chain,” and “food poisoning,” along with the combination of terms such as “heavy metal in Nigeria,” “heavy metal and food chain in Nigeria,” “heavy metals and health in Nigeria,” and “heavy metals and food chain contamination” were applied to the search. Authors adopted the idea by Manikas and Hansen [35], with which they designed and applied a set of five inclusion and exclusion criteria: (1) the literature should address heavy metals as an area of core research, (2) the literature must be a research paper, i.e., being published after a rigorous peer review, (3) the language expressed in the selected article must be English, (4) the literature must not be more than ten years of age from the time of the present research, except when it is a seminal study, and finally (5) the literature must be none of these: an abstract with extended perspective, short communications, letters, and presentations. Although authors relished bringing in the global focus of environmental geochemistry and heavy metal toxicity, they maintained that a good number of the sourced studies must focus on heavy metals in Nigeria. With this aim, the study had to consider some locally based journals in situations where local ideas seemed unassailable and inevitable, but authors made sure that they maintained the same criteria for exclusion and inclusion.

On the whole, studies derived from the literature search have acknowledged the richness and usefulness of heavy metals within the human-ecological system. It also acknowledged significantly the negative implications of these metals, especially in the food chain. Discussions on the toxicity of heavy metals also include the means to assess and mitigate these impacts. Though Kumar et al. [38] discussed the process of phytoextraction, Ali et al. [24] presented phytoremediation of heavy metals. It also shows that other methodologies including photocoagulation [39], biosorption [40], and rhizofiltration [41] have been found in the extant research literature. These are proven scientific techniques that have now formed the basis of best practices in the study of the impacts of heavy metals on the food chain system. Finally, the literature search shows that despite the wide-ranging discussions on heavy metals and food chain, research has developed little regarding the application of more scientific techniques to mitigate the heavy metal impacts on the food chain in Nigeria.

## 3. Results and Discussion

Nigeria is one of the DCs facing massive environmental contamination through natural constituents such as heavy metals and metalloids. The concentration levels of heavy metals alongside their bioavailability and bioaccessibility can cause contamination of trophic levels, as shown in Table 1. This table also shows different regions where foods could serve as good dietary sources due to essential trace metals, besides the levels for human consumption safety margin. Animals, leafy vegetables, and fruits are consumed widely because of their nutritional value and culinary purposes. Therefore, they are part of the daily diet in many Nigerian households. The consumer's perception determines the quality of such food and vegetables. Although, this perception is subjective, it is mostly based on the measure of the colouration and leaf size of vegetables. Hence, there is a general assumption that vegetations with dark green and big leaves are of better quality. However, Mapanda et al. [63] have shown that external morphology is inadequate for leafy vegetables qualitative assessment as heavy metals rank high amongst the major contaminants. In the soil, heavy metal accumulation depends on plant species. However, the efficiency of plants in absorbing metals is determined by either plant uptake or soil-to-plant transfer factors of the metals. Plant tissues absorb and accumulate heavy metals based on the available pH, moisture content, temperature, organic matter, and nutrient [64].  Table 2 shows some risks associated with food chain contamination for humans and some trophic levels in Nigeria.

## 4. Potential Health Hazards of Some Selected Heavy Metals and Metalloids

### 4.1. Lead (Pb)

Toxicological review of lead has shown inhibition of the activity of *d*-aminolaevulinic dehydratase (porphobilinogen synthase, one of the major enzymes involved in the biosynthesis of heme) and developmental problems such as impaired cognitive function, behavioral disorder, stunted growth, and impaired hearing at blood lead level as low as 5 *μ*g/l [9, 78]. Lead also interferes with calcium metabolism, both directly and by interfering with vitamin D metabolism; it concentrated largely in bones in animals and humans and interferes with the normal maturation of erythroid elements in the bone marrow [79]. These effects have been observed in children at blood lead levels ranging from 12 to 120 *μ*g/l. Lead is toxic to both the central and peripheral nervous systems, inducing subencephalopathic neurological and behavioral effects. Other effects include epidemiological effects (blood lead level of 30 *μ*g/l), intelligence quotient deficits of about four points in children due to prenatal/postnatal exposure to lead (blood lead level ranging from 11 to 33 *μ*g/l), headache, irritability, constipation, weight loss, fatigue, hypertension, miscarriages, stillbirths, and renal tumors [80]. However, there is evidence from studies in humans that adverse neurotoxic effects other than cancer may occur at very low concentrations of lead, and that a guideline value derived on this basis would also be protective for carcinogenic effects [81]. A report of prevalence dental caries in children in the Tamar Valley, England, and in Ceredigion, Wales, was associated with a high level of Pb in soils which were available to plants [79].

### 4.2. Manganese (Mn)

Adverse effects of Mn can result from both deficiency and overexposure, causing neurological effects due to high level in drinking water, which can cause tremor, gait disorders (seen in primate), psychological symptoms such as irritability, and emotional liability [80]. For instance, amyotrophic lateral sclerosis, according to Hubbs-Tait et al. [80], is a progressive neurological disorder, which appears to be a disease that may reflect a deficiency of Mg or Mn. The decreased intake of Mg or Mn leads to a decreased ability to store and use thiamin (vitamin B_1_). These authors further noted that there is some evidence suggesting that Parkinson's disease may be casually related to Mn, but in this case, to an excess of it.

### 4.3. Nickel (Ni)

Nickel is an essential trace element in animals. Some of its health risk includes fibrosis, chronic bronchitis, impaired pulmonary function, and emphysema [82]. Allergic contact dermatitis is the most prevalent effect of toxicity of nickel in the general population [78]. However, it is suspected to be an essential element for some plants and animals [83]. According to Plant and Thornton [84] and Carla [85], Ni deficiency results in decreased plasma cholesterol, increased liver cholesterol, ultrastructural changes in the liver calls, rough hair, impaired reproduction, and poor growth of the offspring.

### 4.4. Zinc (Zn)

Zinc is an essential trace element whose threshold value in surface and groundwater normally do not exceed 0.01 and 0.05 *μ*g/l, respectively [86]. However, concentrations in tap water can be much higher as a result of dissolution of zinc from pipes. It should be noted that drinking water containing zinc levels above 3 *μ*g/l may not be acceptable for consumers [87].

### 4.5. Cobalt (Co)

Cobalt is an essential microelement for humans in the form of vitamin B_12_ with a complex pathway through the food to man [79, 86]. Deficiency of Co results in pernicious anemia syndrome, whereby the intrinsic factor from the stomach that facilitates B12 absorption is absent, characterized by larger than normal (macrocytic) red blood cells plus neurologic abnormalities. Excessive Co added to the foam stabilizer in beer however produced severe cardiomyopathy, haematologic, neurologic, and thyroid abnormalities in humans [79, 88]. A relationship between Co/iodine (I) ratios in geochemical environment showed an inverse correlation between Co in water and soil and thyroid enlargement in animal and man.

### 4.6. Copper (Cu) and Chromium (Cr)

These are important essential elements but when consumed in excess, they cause toxicity [89, 90]. It should be noted that the threshold for the effects of copper on the gastrointestinal tract still leaves some uncertainty regarding the long-term effects of Cu on sensitive populations, such as carriers of gene for Wilson disease and other metabolic disorders of copper homeostasis [81]. Copper deficiency results in kinky and steely hair syndrome in humans and abnormal wool in sheep, while excessive Cu intake results to hepatolenticular degeneration with progressive impairment of Cu-laden tissues until death results [81]. It also helps in interconversion of the major neurotransmitters, dopamine, noradrenaline, and adrenaline, and in pigment production. Zinc-Cu interaction has shown hypothesis of ischemic heart disease, which proposes that decreased Cu intake with excessive Zn may play an aetiologic role in cardiac deaths in both animals and man [79, 88]. Contamination and pollution of vegetable and soils near smelters does occur, and excessive Cu in drinking water has been reported to have caused a toxic syndrome in an infant called pink disease [84]. Chromium helps to maintain blood glucose levels, but its toxicity can result in allergic dermatitis such as eczema [91].

### 4.7. Cadmium (Cd)

Cadmium may also be adsorbed onto organic substances, such as humic and fulvic acids, and therefore organic-rich waters may have higher Cd concentrations given a local Cd source [79, 85]. Chronic exposure to the metal can lead to kidney disorders, anemia, emphysema, anosmia (loss of sense and smell), cardiovascular diseases, renal problems, and hypertension [84]. Itai-itai disease appears to be a Cd-related disease, which is very painful and causes the wastage and embrittlement of bones [78].

### 4.8. Arsenic (As)

Arsenic is a metalloid whose chronic exposure effects include tingling, numbness, and peripheral neuropathy according to Plant and Thornton [84]. These authors also argued that arsenic toxicity in cattle has been found to cause dysentery and respiratory distress. An ecological correlation between the arsenic level of well water and mortality from various malignant neoplasm in China (Province of Taiwan) demonstrated a significant association with the arsenic level in well water ranging from 0.35 to 1.14 mg/l with a median of 0.78 mg/l for cancer of the liver, nasal cavity, lung, skin, bladder, and kidney and hyperpigmentation, hyperkeratosis, Blackfoot disease (a type of gangrene) in both males and females, and prostate cancer in males [92].

Heavy metal contamination is a global issue [93–97] and of high significance in Nigeria. Evidence from research within the context of Nigeria suggests a growing concern for protecting different varieties of food from the toxic effects of heavy metals [30, 69]. The present outcome from various laboratory investigations, such as those of Ogunkunle et al. [56] and Obiora et al. [29], show that some of these heavy metal contaminations, particularly those affecting dairies, most of the aquatic habitat, and the beverage industry, are within safety level, according to international standards. However, chances are that they would heighten their toxicity because of the ever-increasing industrial and anthropogenic activities. Therefore, future toxicology assessment and food chain safety measures are being recommended [62].

Of all the heavy metals found in the literature, Pb, Cd, Hg, and Mn have the highest health risk index (HRI) in Nigeria because of their bioavailability. These heavy metals are present in most of the staple foods, such as cassava found in the southern and southeastern parts of Nigeria. This is a risky situation for people who live in these areas where the consumption of such foods is part of the endeared human culture. Other findings and concerns from the review include the following:

1. Localised research, that is, state-based studies by previous authors. Localisation of study areas tends to reduce bias as data and validation for research on a country-wide scale could increase uncertainties which might complicate important issues.
2. Heavy metal concentrations found in beverages, bouillon cubes, and food condiments in Nigeria are within the EU/WHO/FAO maximum permissive limits, and hence pose no health risk [44, 62]. However, those in some varieties of fish (*Clarias gariepinus*), cassava, fruits, and vegetables in different regions of the country show significant potential health risk.
3. The main pathway through which these heavy metals contaminate the food chain in Nigeria is the soil, water, and other inorganic substances by the process of leaching and runoff while human exposure pathways to these heavy metals are ingestion, inhalation, and dermal [30]. Foods on sale in the open marketplace are at high risk of being contaminated by heavy metals [56].
4. In the global literature, several scientific techniques are applied to reduce the toxicity of heavy metals in the food chain. Dash et al. [98] presented rhizobacteria which the authors argued was most suited for alleviating the stress of heavy metals in the agroecosystem. Li et al. [99] discussed a number of techniques involving chemical, physical, and biological methods including phytoremediation as a cost-effective, environmentally friendly, and aesthetical measure. Evidence from the literature shows that these techniques are lacking in Nigeria. However, a few primordial and conventional toxicity reduction techniques are recorded, and this include education of farmers to reduce the use of pesticides [100] and proper disposal and siting of municipal wastes [101].

## 5. Conclusion

Heavy metal poisoning of the food chain has stimulated increasing research interests within the field of environmental chemistry and toxicology. Earth materials comprise an abundance of heavy metals that support the ecosystem processes. Several cases of human health impacts have resulted because of bioavailability, bioaccessibility, and excessive concentrations and distribution of contaminants in the food chain. These heavy metals contaminate most of the soils, farmlands, and aquatic habitats. The chemistry of these metals, rather than their density, is a major contributor to their usefulness, more so their negative impacts on the human-ecological systems.

Although existing research on food chain contamination in Nigeria is fragmented and localised, this review has shown that heavy metal contamination affects Nigeria's food chain although at present, the amount of these toxic substances found in beverages, food condiments, and some aquatic animals is at a safety level. Sources of contaminants are both natural and anthropogenic. These toxic substances make their way to the farmland, water, and food stuff exposed in the market by mostly the process of leaching and runoff. Anthropogenic activities contaminate different environmental media such as the soil, water, and air which support the growth, production, and supply of foods. Ingestion, inhalation, and dermal are the major exposure pathways through which heavy metals in locally produced foods in most of the regions get to the consumers. Toxicity of these heavy metals can cause potential health hazards. Thus, long-term consumption of these foods is of public health significance. There is the need for better quality control for food crops to protect consumers from contamination exposure. In addition, close monitoring of the significant possibility of significant harm of these heavy metals on the health of the population in these regions is essential. It is also important to use remediation measures in contaminated areas where local foods are grown or produced.

In view of the implications of heavy metals in the food chain in Nigeria, researchers showed insufficient fundamental discussions on the understanding of the true nature of the health risk to inform practical and implementable decision and policy. It therefore shows that a country-wide knowledge base is lacking. This is a major limitation, which possess potential constraints on insight for future research focus. Therefore, this prevention study aimed to address the gaps in knowledge by synthesising existing knowledge and drawing from the examples presented in extant research. In addition, this review shows that more scientific techniques for mitigating the impacts of these heavy metals' contamination are lacking in Nigeria although research documents a few primordial and conventional toxicity reduction techniques. The spatial distribution of these toxicities in Nigeria's food chain is the major issue that suggest the need for further research, which should map and delineate spatially the variations in heavy metal toxicity within the whole of Nigeria. This will provide insight into those states and localities that mostly need food intervention. It will further enhance quality decision and policy making towards country-based food safety adaptation and capacity to cope with food toxicity and diseases resulting from heavy metal contamination of the food chain.

## Figures and Tables

**Table 1:** Regional environmental contamination of some selected trophic levels in Nigeria.

Region	Some selected trophic level			Author(s)
	Producers (plants/crops)	Herbivores	Carnivores	
East Examples: Pb, Cd, and Ni concentrations in food crops and fruits in Owerri, Imo State, exceeded maximum acceptable levels for agricultural soil Potential lead contamination in food crops in Anambra and Ebonyi States Traces of heavy metals were found in bouillon cubes and food condiments in Umuahia, Abia State Pb-contaminated cassava tuber and lemon grass and Mn-contaminated leafy vegetable in Enyigba, Ebonyi State	*Oryza sativa, Glycine max, Pentabacta microfila, Canarium schweinfurthii, Citrus reticulate*, and *Ananas comosus* were contaminated by Pb, Cd, and Ni in soil in Owerri. *Oryza sativa* in soil in Enugu State showed a Pb and Cd total hazard index of >1. Studies have also revealed Pb exceedance in *Prosopis africana, Xylopia aethiopica, Piper gineense, Monodora myristica*, and *Capsicum frutescens*. Others include cassava and lemongrass	Goat, rodents, cattle	Fish, crabs, bird, bear	Orisakwe et al. [31]; Ihedioha et al. [9]; Asomugha et al. [42]; Okoronkwo et al. [43]; Nnorom et al. [44]; Obiora et al. [29]; Ihedioha and Okoye [45]

South Examples: green vegetables showed higher concentrations of heavy and trace metals than other crops in Rivers State. Hart et al. [46] posted higher concentrations of Zn, Cd, Pb, Fe, Cu, Cr, Co, and Mn in crops grown in oil-producing areas than those from crops in nonoil-producing areas	Okra and cocoyam with 9.1 mg/kg and 1.1 mg/kg, respectively, in oil exploration sites in Rivers State. Other crops include cassava and plantain, which showed higher concentration of Cr, Cu, Pb, Fe, and Zn around Etelebou Oil Flow Station in Bayelsa State, indicating long-term accumulation of these heavy metals in soil	Goat, cattle, ram	Fish, periwinkle and other sea foods, bird. Heavy metals in fish (*Clarias gariepinus*) organs from Asaba major markets, Delta State, Nigeria	Kelle et al. [47]; Hart et al. [46]; Worgu [48]; Alum et al. [49]; Nkwocha et al. [50]; Nkpaa et al. [51]

West Examples: higher heavy metal concentrations in *Amaranthus* grown on Lagos highway soil than in those from controlled soil. Higher concentration of heavy metals and metalloids in the plant organs cultivated on Ekiti dumpsites in addition to seasonal variation of heavy metal concentration levels (suggesting element mobility heavy metal adsorption by plants)	*Amaranthus*, groundnut, maize grains, spinach, roselle leaves, okra, onions. In Ekiti, Ni concentration level in okra reduced by 45.19% in rainy season while Fe by 8.25%. There is also potential contamination of Pb, Zn, and Hg in these vegetables	Cattle, ram, goat,	Fish, bird, bear	Nduka et al. [52]; Adeyeye [53]; Zhou et al. [54]; Olayiwola et al. [55]; Ogunkunle et al. [56]

North Examples: heavy metals such as Pb and Cd have been found to be highly toxic (exceeding the World Health Organisation (WHO) and the Food and Agriculture Organisation (FAO) maximum permissive limits) in farms in Kaduna and Kano metropolis. Higher heavy metal concentration in soils from the Itakpe iron ore mining site than those from controlled soil in Kogi State. Some of the heavy metals in both mining and nonmining sites had concentration below the WHO/FAO maximum permissive limit for food	Contaminated spinach, jute mallow, and tomatoes in Kaduna and Kano farms by Pb and cadmium. Permissive concentration levels of Cu/Zn, respectively, in peppers (5/13.5 mg/kg), tomatoes (5.5/15.5 mg/kg), and onions (4/16.75 mg/kg) in soils at Itakpe	Cattle, ram, goat, donkey	Bird, panda, catfish (UKE Stream in Nasarawa State), *Clarias* and tilapia in Lake Geriyo, Adamawa State.	Bawuro et al. [57]; Jacob and Kakulu [58]; Matthews-Amune and Kakulu [59]; Iyaka [60]; Opaluwa et al. [61]

A general review of heavy metal concentration and potential health implications of beverages consumed in Nigeria. Heavy metal concentrations, including iron, mercury, tin, antimony, cadmium, zinc, copper, chromium, lead, and manganese, seldom exceed the maximum contaminant level recommended by the Standard Organisation of Nigeria (SON) and the World Health Organisation (WHO) as applicable to drinking water resources				Izah et al. [62]

**Table 2:** Risk/implications of food chain contamination for humans, domestic life, and wildlife in Nigeria.

Region	Common heavy metals	Major sources	Risk/implications	Author
East	Pb, Al, Zn, Cr, Fe, Mn, Cu, Co, As, Ni, Hg	Heavy metals and metalloids from municipal and industrial wastes in Onitsha/Awka and Abagana in Anambra and Abia States, respectively, mining and quarrying (e.g., Pb-Zn mining and igneous rock quarrying and limestone mining at Ishiagu and Nkalagu, respectively, in Ebonyi State), air pollution from transportation, agrochemicals	Potential heavy metal toxicity in the body system that can lead to hemoglobinuria, gastrointestinal disorders, ataxia, pneumonia, diarrhoea, stomatitis, and paralysis	Orisakwe et al. [31]; Ihedioha et al. [9]; Asomugha et al. [42]; Odika et al. [7]; Jaishankar et al. [65]; Nduka et al. [52]

South	Zn, Cd, Pb, Fe, Cu, Cr, Co, and Mn	Petroleum extraction and refineries, e.g., consumers of seafood from these contaminated sites in Ogoniland may be exposed to metal pollution	Environmental degradation resulting to excessive social unrest and destruction and incessant harm to the physical, social, environmental, and economic health of its inhabitants	Nkpaa et al. [51]; Worgu [48]

West	Pb, Al, Zn, Cr, Fe, Mn, Cu, Co, Mo, Se, As, Ni, Hg	Aerial deposition by emissions from Lagos traffic, leaching and runoffs of contaminants from Ekiti State dumpsites. Contaminated freshwater bodies in Osun State. Accumulation of heavy metals from battery waste in topsoil, surface water, and garden grown maize at Omilende area, Olodo, Nigeria	Long-term consumption of (leafy) *Amaranthus* from Lagos highway soils poses potential health risk due to its heavy metal concentrations. Health risk index (HRI) and daily intake of metal (DIM) estimation indicate higher health risk indexes in vegetables containing Pb, Ca, and Hg. Osun freshwater bodies showed high carcinogenic risk from high concentrations of heavy metals	Atayese et al. [66]; Nduka et al. [52]; Adeyeye [53]; Balkhair and Ashraf [67]; Titilawo et al. [68]; Afolayan [69]

North:	Pb, Zn, Cr, Fe, Mn, Cu, Co, Ni,	Some heavy metals are introduced into the environment from farmyard and chemical fertilizer application (agrochemicals). Others include domestic by-products, worn automobile tires, roofs, brake linings, and waste food. There are also iron mining sites (Itakpe, Kogi State). Sample of tested sediment from the river Ngada, Maiduguri in Borno State revealed provocative amounts of heavy metals. Evidence of mobility of heavy metals from dumpsites to farmlands through leaching and runoff in Lafia, Nasarawa State	Increase concentration of zinc in pasture fields. Anthropogenic sources usually produce heavy metals that are high in instability and solubility and in turn, result in high bioavailability which increases potential health risk. Decrease in soil productivity by high Pb concentration, while extreme low concentration can inhibit some essential plant processes such as mitosis, water adsorption, and photosynthesis. This can lead to brown short roots and stunted growth/foliage. Toxicity of heavy metals on the other hand can lead to reduced ability in leguminous plants to fix molecular nitrogen	Bawuro et al. [57]; Agbenin [70]; Anguelov and Anguelova [64]; Armah et al. [71]; Tangahu et al. [72]; Khan et al. [73]; Bhattacharyya et al. [74]; Guala et al. [75]; Singh et al. [76]; Akan et al. [77]; Opaluwa et al. [30, 61]
